# Empowering community control over alcohol availability as a suicide and self-harm prevention measure: Policy opportunity in Aotearoa New Zealand

**DOI:** 10.1016/j.lanwpc.2022.100631

**Published:** 2022-11-24

**Authors:** J. Boden, S. Hetrick, N. Bowden, S. Fortune, L. Marek, R. Theodore, T. Ruhe, J. Kokaua, M. Hobbs

**Affiliations:** a Christchurch Health and Development Study, University of Otago – Te Whare Wānanga o Ōtākou, Christchurch, Canterbury, New Zealand; b Department of Psychological Medicine, University of Auckland – Waipapa Taumata Rau, Auckland, New Zealand; c Centre for Youth Mental Health, University of Melbourne, Melbourne, Australia; d A Better Start: E Tipu e Rea National Science Challenge, New Zealand; e Department of Women's and Children's Health, University of Otago – Te Whare Wānanga o Ōtākou, Dunedin, New Zealand; f School of Population Health, University of Auckland – Waipapa Taumata Rau, Auckland, New Zealand; g Faculty of Health, University of Canterbury – Te Whare Wānanga o Waitaha, Christchurch, Canterbury, New Zealand; h Te Taiwhenua o te Hauora – GeoHealth Laboratory, University of Canterbury – Te Whare Wānanga o Waitaha, Christchurch, Canterbury, New Zealand; i National Centre for Lifecourse Research, University of Otago - Te Whare Wānanga o Ōtakou, Dunedin, New Zealand; j Division of Health Sciences, Va'a O Tautai-Centre for Pacific Health, University of Otago - Te Whare Wānanga o Ōtakou, Dunedin, New Zealand; k The Cluster for Community and Urban Resilience (CURe), University of Canterbury – Te Whare Wānanga o Waitaha, Christchurch, Canterbury, New Zealand

One of the most pressing issues in public health in Aotearoa New Zealand (NZ) is our rate of suicide and self-harm, particularly among young people.^1^^,^^2^ The 2018 New Zealand Government Inquiry into Mental Health and Addiction recognised the challenge of reducing these rates, and raised the important issue of the role of alcohol and other substance use in increasing suicide risk.^1^

The link between alcohol and suicidal behaviour is well established. Internationally, acute alcohol use is associated with 10–69% of suicides^3^; NZ is no different. Among 4658 suicides in those aged 15 years or over between July 2007 and December 2020, 26.6% involved heavy alcohol consumption.^4^ Three aspects of alcohol consumption are pertinent to suicide prevention.^5^ Firstly, alcohol intoxication has a disinhibiting effect, where peoples’ decisions or actions regarding suicidal distress may vary from decisions or actions they would have taken if not intoxicated. Secondly, there are detrimental impacts of binge or disordered drinking on poor mental and physical health, both of which are associated with elevated risk of suicide. Thirdly, the deleterious effect on children and young people exposed to hazardous alcohol use by adults in their lives is an important aspect of suicide prevention.^6^

Self-harm (intentional self-poisoning or self-injury regardless of degree of suicidal intent) is associated with a greater risk of all-cause mortality and suicide,^7^ and is particularly concerning as suicide is a leading cause of preventable death in young people in NZ.^8^ International evidence demonstrates those who reported self-harm at age 16 were nearly twice as likely to report harmful alcohol use at 18 years^9^ and that heavy episodic drinking is associated with increased risk of self-harm.^10^ In NZ, longitudinal data shows that alcohol use disorder over the lifecourse predicts suicidal ideation.^4^

Nationally representative NZ data shows proximity to alcohol outlets is associated with increased hazardous drinking and crime.^6^ Alcohol outlets are more often located within specific geographical areas unfairly targeting more deprived areas of NZ.^6^ Reducing alcohol availability is a well-established policy mechanism to reduce alcohol consumption, which could result in reduced alcohol-related self-harm and suicide and reduce health inequities.

The Sale and Supply of Alcohol (Harm Minimisation) Bill introduced in June 2022^11^ seeks to reduce alcohol-related harm in NZ through two well-researched means.^5^ First, it limits the advertising of alcohol products at sporting events (including broadcasts of sporting events) and prohibits alcohol industry sponsorship of sports (see Chambers et al.^12^ (2021) for a detailed discussion of the merits of this approach). Second, and the focus of this comment, it empowers local communities to have more control on alcohol availability. Under existing legislation, local councils can develop Local Alcohol Policies (LAPs) to further community involvement into the provision of alcohol in their areas. LAPs can specify the number (if any) and location of new alcohol outlets in the community, as well as the hours and conditions (e.g. storefront advertising) of sale.^13^ However, LAPs are often blocked by large companies using their right of appeal. Most councils do not have the resources to undertake the ensuing lengthy legal battle and therefore opt to appease the alcohol industry by weakening the protections in their LAPs.^14^ Others, such as the Auckland Council, continue to engage in lengthy legal proceedings, costing millions, and in the meantime, their communities pay the price, without any LAP protections in place. Thus, community attempts to influence the location and density of alcohol outlets have been rendered ineffective.^11^ Enabling local public health approaches to alcohol represents a major step to realising tangible reductions in alcohol related harm in NZ.

Self-harm and suicide are complex issues, with a myriad of causes. However, it is clear that reductions in problematic alcohol consumption is an important, evidence based suicide prevention strategy.^15^ Given the emphasis on reducing suicide rates in NZ, any policy and law change that will reduce alcohol consumption in the population is likely to provide benefit in this regard. For many communities, Māori, Pacific or those with limited socioeconomic leverage, restricting alcohol outlets and consumption may provide a useful tool for those wanting to improve mental wellbeing and reduce rates of self-harm and suicide. We therefore support the passage of the Sale and Supply of Alcohol (Harm Minimisation) Bill, and we look forward to working with this and future Governments to reduce the harms to mental and physical health posed by alcohol.

## Declaration of interests

None.
